# Emerging infrastructures: the politics of radium and the validation of radiotherapy in India’s first tertiary cancer hospital

**DOI:** 10.1057/s41292-020-00223-3

**Published:** 2021-03-05

**Authors:** Robert D. Smith

**Affiliations:** Department of Anthropology and Sociology, Graduate Institute of International and Development Studies, Chemin Eugène-Rigot 2A Case Postale, 1672 1211 Geneva 1, Switzerland

**Keywords:** History of medicine, History of cancer, History of radium, Philanthro-capitalism, Post-colonial science and medicine, India, Nationalism

## Abstract

This article traces the history of India’s first tertiary cancer hospital, Tata Memorial Hospital (TMH). TMH was originally conceived in 1932 as a philanthropic project by the Tatas, an elite Parsi business family in Bombay. The founding of TMH represented a form of philanthro-capitalism which both enabled the Tatas to foster a communal acceptance for big businesses in Bombay and provide the Tatas with the opportunity to place stakes in the emerging nuclear research economy seen as essential to the scientific nationalist sentiment of the post-colonial state. In doing this, the everyday activities of TMH placed a heavy emphasis on nuclear research. In a time when radium for the treatment of cancer was still seen as ‘quackery’ in much of the world, the philanthro-capitalist investment and the interest in nuclear research by the post-colonial state provided an environment where radium medicine was able to be validated. The validation of radiotherapy at TMH influenced how other cancer hospitals in India developed and also provided significant resources for cancer research in early-mid twentieth century India. Ultimately, this article identifies ways in which cancer comes to be seen as relevant in the global south and raises questions on the relationship between local and global actors in setting health priorities.

## Introduction

In the global south, the provision of cancer care is often framed as inadequate due to a lack of resources and capacity. In this article, I provide a novel perspective on how cancer care is able to be made relevant as a public health priority and how cancer therapies come to be seen as valid biomedical treatments in the global south. To do this, I trace the history of India’s first tertiary cancer hospital, Tata Memorial Hospital (TMH), which was founded in Bombay in 1941 by the philanthropic Tata Trust. However, I show that the Tata Trust’s decision to found TMH was motivated beyond altruism. When founded, the Trust planned and operated TMH with a specific focus on the research and development of radium; the Trust’s focus on radium represented a form of philanthropic capitalism which enabled the Tatas to place stakes in the emerging interests of the nuclear research economy (Vevaina 2018; TMC 2017). In a time when the use of radium for the treatment of cancer was often viewed as ‘quackery,’ I argue that it was the promise of radium to fulfill the nuclear research desires of post-colonial scientific nationalism that enabled radium-based cancer therapies to be seen as a valid form of treatment at TMH (Cantor 2008; Phalkey 2013). The use of radium for cancer treatment was used to support a form of nation building which paradoxically emphasized the potential of radium while showing little ethical concern for its lack of therapeutic efficacy. The history of TMH evidences how the emergence of cancer infrastructures in the global south are more than systematically planned health systems responses, but are highly contingent upon local and global politics, capitalist potentials, and nationalist ideologies.

This article begins by outlining the recent work of social science in adding to the understandings of cancer in the global south. I then go on to discuss the role of philanthropy in providing health care in Bombay before narrowing the discussion to detail the burden of cancer and its common therapies in the early-mid twentieth century. Finally, drawing upon extensive archival sources, I trace the actors, institutions, and interests within philanthro-capitalism and post-colonial scientific nationalism which led to the founding of TMH and the legitimization of radium as a form of treatment.

## The role of social science in understanding cancer in the global south

A growing body of social science literature has emerged as cancer burdens have increased in the global south. Authors have noted how medical and public health literature often focus on abstract health systems discussions at the expense of accounting for the complexities of the lived experiences of cancer patients; complimenting this work, the role of social science is then to “emphasize local conditions; question basic assumptions; and call for social, cultural, and economic changes that are politically challenging” (Caduff et al. 2018, p. 7). Specifically, methods of ethnography and historiography have begun to fill these gaps by documenting the responses of patients, physicians, and institutions to the complexities of cancer in the global south. These understandings are important to illuminate the gaps between the theory and practice of oncology and are able to reveal what cancer ‘therapy’ means in contexts mediated by often contradictory interests of social, political, and economic actors.

Ethnographers’ deep engagement with the life worlds of cancer patients reveals the unique ways in which cancer patients understand and make meaning out of cancer in the global south. Julie Livingston’s (2012) seminal ethnography of a Botswanan cancer ward reveals how a lack of biomedical capacity forces oncologists to ‘improvise’ therapy by stitching together available resources. Patients’ ability to understand and make meaning of cancer becomes embedded in the sites where oncologists are ‘improvising medicine.’ Further, Dwaipayan Banerjee’s (2020) recent book, *Enduring Cancer,* documents cancer patients in Delhi and provides gripping detail of how the meaning of cancer “was to be newly awakened to the fragility of social ties” as much as the social ties of a patient were awakened to cancer (3). For Banerjee, “the force and impact of a cancer diagnosis” was textualized by the “older cracks and fault lines” within the patient’s life world (3). Banerjee’s ethnography testifies to how a patient’s meaning of cancer then goes beyond the disease itself and as such the practice of oncology. Similarly, Djordjevic (2019) documents how making meaning of cancer in Rwanda must take into account a double burden of communicable and noncommunicable diseases, cancer’s “etiological uncertainty … and [a] frequent resistance to treatment” (553). In a context where biomedicine appears insufficient, an epistemological contradiction arises whereby patients find meaning in cancer through the “efficacy of occult acts” while oncologists falsely assume patients “to quickly relinquish any beliefs in the occult and fully embrace a biomedical value system” (ibid). Oncologists’ commitment to biomedical epistemologies originating in the global north then “limits the ability” of the practice of oncology to acknowledge “the limits of treatment” and more comprehensively alleviate suffering.

These findings reveal that when utopic global north visions of biomedical practice are transplanted, they often exist in an epistemological incommensurability with the unique needs produced through the life worlds of cancer patients in the global south. This has frequently opened up new sites for institutions to respond to patients’ needs. Banerjee (2019) has identified one such example of a philanthropic cancer palliation service in Delhi. He emphasizes the pastoral logic of these institutions whereby their attempt to take on the surplus needs of cancer patients are institutionally confined to “a single mandate” for purposes of sustainability (ibid, 6). In this sense, even institutions which seek to bridge the epistemological gaps between transplanted global north oncology practices and the patient needs of the global south still fall short. This literature ultimately shows how the complexity of this epistemological gap makes the unique needs of cancer patients in the global south ‘oblique’ unless dissected through forms of deep ethnographic engagement (Caduff et al. 2019).

While ethnography lays bare the navigations of cancer patients and other stakeholders within this epistemological gap, it does not reveal the *how* and the *why* in which oncology came to inhabit this space in the global south. Adding to the ethnographic literature, historiography has taken up these questions. Lochlann Jain’s (2013) interdisciplinary ethnographic and historical analysis of cancer in the USA shows how cancer was “anything *but* an objective thing, cancer can be better understood as a set of relationships—economic, sentimental, medical, personal, ethical, institutional, statistical … [and for these reasons,] we desperately need new ways of understanding cancer” (4). Jain’s analysis has ruptured teleological claims that oncology has existed in a continuum of progress, and instead calls us to ask how cancer has come to legitimize itself as an ‘objective thing’ (also see: Mukherjee 2012; Timmerman 2013; Löwy 2011; Weisz 2014). Historians of medicine who analyze cancer in the global south emphasize how the legitimization of cancer therapy takes place between highly localized contexts constituted of social, economic, and political actors and reigning biomedical paradigms. Importantly, the legitimization of cancer therapy has rarely been exclusively motivated by the burden of cancer nor the needs of cancer patients.

Kavita Sivaramakrishnan (2019) documents how the founding of India’s third tertiary cancer hospital in Madras in 1954 was embedded in local politics requiring activists to overcome Indian leaders’ perceptions which viewed cancer “in terms of ‘difference,’ as a state of exception … and [a disease which] merited concern, but not sustained commitments” (5). The success of activists in receiving public land and funding was not because of cancer being “seen, reported and experienced in debilitating ways among individuals and local communities,” but instead through activists’ ability to draw together the interests of “middle-class women, urban philanthropy, and … male political leaders, and health officials” in a way which ‘irritated’ the state (ibid, 5, 1). To do this, activists’ narratives were required to be “ambivalent, and even contradictory:” they drew attention to the “ubiquity of social suffering” while placing special emphasis on “feminized social metaphors for care;” emphasized the “deaths of middle-class leaders and loss of productive citizens” while also stressing “the moral obligations of the state” to all vulnerable citizens; even advocating for “individual responsibility through education and self-screening” while simultaneously evoking the urgency of cancer’s “chronic and fatal morbidity that was mostly detected too late to treat” (ibid, 6).

Lucas Mueller’s (2019) historiography of cancer research from 1920 to 1960 in Kenya further adds to our understandings of how cancer research was modified through time by global biomedical paradigms to maintain legitimization. Mueller shows how the disciplinary shift from geographic pathology to epidemiology in researching cancer “was intended to benefit European and North American scientists at a time of growing alarm over increasing rates of cancer” (Caduff and Van Hollen 2019, p. 4). This disciplinary shift further “signaled a shift in research focus, from one dedicated to diagnostics and the environment to one centered on population and statistical studies” (Mueller 2019, p. 1). These shifting ideas of legitimate cancer research had downstream effects on what types of cancer infrastructures were able to be built. Mueller’s findings demonstrate that “it was not the lack of knowledge about cancer in the developing world but rather specific configurations of knowledge that shaped which cancer interventions in the developing world researchers and public health officials conceived” (ibid).

Marissa Mika’s (2017) historiography of a Ugandan cancer center shows how international collaborations brought “vital yet partial investments in improving the capacity of medical facilities” (1). In what may otherwise be characterized as eclectic investments, Mika shows how these capital flows were “tied … to shifting international research priorities … [and] broader upheaval and periods of stability in Uganda” (ibid). In this sense, Mika’s case occupies a middle ground showing how international priorities intersected with local politics. These global and local intersections evidence how “extreme oscillations” of international research investment were able to “shape a culture of care and oncology practice that lived on” (ibid).

What these historians of medicine show is *how* historically competing political-economic interests have shaped cancer infrastructures in the global south, and as such *why* cancer patients must navigate their life worlds today within restricted conditions of possibility. In this article, I bring another perspective to this discussion by documenting the founding of India’s first tertiary cancer hospital, TMH, from the time period of 1932 to 1963 in Bombay. What is at stake in this historiography is twofold: first, I document how cancer and philanthropy intersected and I show how these interests generated forms of capitalist power; second—differing from Sivaramakrishnan’s case which battled against the state and Mueller’s case which ceded to the biomedical paradigms of the global north—I show how the Tatas and the Government of India’s (GOI) mutual interests in nuclear research made possible the use of radium for the treatment of cancer at a time when the global biomedical paradigm largely rejected its therapeutic efficacy. To begin this history, I will start by detailing the political history of therapeutic radium to illuminate the stakes of radium in the twentieth century.

## Radium for the treatment of cancer in the early twentieth century: India and the world

The first “therapeutic use of radiation appeared in March 1897,” and the practice of radium for treating cancer largely began in the early twentieth century (Hayter 1998, p. 2). The legitimization of radium as a form of cancer treatment took place between competing disciplinary specializations in oncology, interwar and cold war politics, and ethical conflicts when little information—yet still significantly some—was known about radium’s harmful effects.

Until the 1910s, the use of radium for therapeutic purposes was commonly viewed as ‘quackery’ because little was known about its efficacy and mechanism of action (Womack 2020). As radium was slowly integrated into professionalized medicine, existing medical disciplines were hesitant to the idea that radium should be a specialty in its own right. For example, Van Helvoort (2001) describes how, in Germany in the 1920s, a significant amount of “infighting” took place between physicians. “Radiotherapy took the form of a struggle … for jurisdiction over” the cancer patient between radiotherapists and surgeons (ibid, 34). In contrast, in the 1920s in England, Ornella Moscucci (2007) documents how female surgeons embraced radium as a form of “feminist opposition” (139). Female surgeons’ use of radium for cervical cancers provided “access to the medical profession in the face of male exclusion from training posts” (ibid). Nonetheless, in a time when medicine was largely dominated by white, male surgeons, the majority saw radium as threatening to the increasingly challenged dominance of surgery in treating cancer; however, these divergent reactions of surgeons also suggest that the evaluation of radium’s efficacy and mechanism of action were equally influenced by emerging professional and social interests.

Beyond the clinic, radium was a highly politicized element linked to inter and cold war tensions making radium challenging to procure. In the early twentieth century, access to radium was scarce, often taking place between government and university laboratory collaborations, and was textualized by the fears of radium’s potential use in war. For example, Angela Creager (2009) traces how conducting radium research in the early twentieth century United States required rupturing “the popular perception that nuclear physics research was unavoidably related to atomic weapons” (222). To do this, American researchers and politicians invested in radium research relied upon the idea that “biomedicine [was] perceived as inherently civilian and physics and engineering as military” (ibid). Thus, this led American stakeholders “to prioritize medical therapy and biological research” in the public representations of their radium research (ibid). However, by 1949, in the context of Soviet Union atomic testing, radium “became entangled in the politics of national security” temporarily limiting civilian availability to radium as it increasingly became “insinuated that the sharing of nuclear materials … was equivalent to the dissemination of nuclear information” (ibid 220). As the USA receded from the global provision of radium, other countries entered the market including the United Kingdom and Canada (Creager 2015). Importantly, these political fluctuations dictated the global availability of radium and slowed the progress which could be made in understanding radium’s therapeutic efficacy and mechanism of action.

In India, the use of radium for the treatment of cancer echoed similar contexts of competing disciplinary specializations in oncology and challenges in procuring radium. Radium was first used as a treatment for cancer in 1913 when the British “Colonel Vaughan of the Indian Medical Service [IMS] had brought radium to Ranchi” (Kavadi 2019, p. 1). The British’s motivations in bringing radium to India is currently unknown; however, the lack of evidence of radium for the treatment of cancer globally suggests that the British’s reasoning was likely beyond the purpose of efficacious cancer treatment. By 1926, radiotherapy—the use of X-rays for therapy—and brachytherapy—the direct application of radium seeds to the cancerous site—were both in practice at the Calcutta Medical College (Banerjee et al. 2014). In 1930, Patna had been designated as a radium facility and began dispensing radium to medical colleges. By 1941, there were four medical colleges in India using radium for the treatment of cancer including Calcutta, Madras, Agra, and Lahore (Kannan and Bajpai 2016). When I interviewed the grandson of Dr. K. M. Rai, the first radium practitioner in Madras, he explained how Rai faced similar disciplinary challenges to those seen globally. When Rai attempted to introduce brachytherapy, he had to “battle” with surgeons to receive access to patients. Further, the prevalence of cancer in India was widely disputed and often went undetected due to a lack of oncological capacity. Most studies of cancers in colonial India were conducted by the British ran IMS and focused on cancers unique to India and specific to poor socioeconomic conditions (Smith and Mallath 2019). Contrasting with epidemiological studies by Indian physicians, the IMS held the “prevailing idea that cancer was rare among the natives of India’ (ibid, 6). In a global context where the supply of radium was hard to come by, this meant that there was little motivation to navigate the global politics of radium to procure a consistent supply.

Globally, radium’s attempt to become accepted within professionalized medicine and its variable supply due to its politicization often enabled physicians to “experiment” with radium on patients (Hayter 1998, p. 2). These experiments were important to medical and national stakeholders because, on the one hand, they fostered biomedical ‘progress,’ while, on the other hand, radium’s therapeutic applications framed it as peaceful and assisted the global depoliticization of radium. However, historians of medicine have shown how these radium experiments had extensive side effects, were at times deadly, and were often performed on vulnerable populations. Ilana Löwy (2012) shows how physicians in France and the USA between 1919 and 1939 “believed that patients should accept ‘everything’ (that is, harsh mutilating therapies)” (104). In this case, radiotherapy “was considered a purely palliative approach … [and only] was proposed to patients who could not be helped by surgery” (105). In Canada, Charles Hayter (2003) shows that from 1933 to 1940 “despite contemporary reports of harm” from radium, radiotherapists bypassed the regulated distribution of radium to treat willing patients (75; also see: Hayter 2005). In the 1940s, American physicians began to die from administering radiotherapy and dangerous patient side effects became widely reported (Sansare et al. 2011). Following this, the use of radiotherapy in the United States largely declined and patients again relied upon surgical treatment for cancer (Mukherjee 2012). However, by the 1960s, the USA Department of Defense had funded total body irradiation trials at the University of Cincinnati which again reported harmful side effects and the frequent deaths of patients (Kutcher 2011).

These histories of radium show that radium’s use was often motivated far beyond hopes for its therapeutic efficacy, especially at a time when it remained ethically ambivalent if its therapeutic effects compensated for its dangerous side effects. Further, in India, physicians were similarly hesitant to accept radium, acquiring radium required large capital investments to sustain a tertiary cancer hospital, and there was a lack of oncological capacity alongside the disputed prevalence of cancer. These circumstances raise the vital question of why the Tatas decided to invest so heavily in radium as they deliberated funding TMH. As we will see, the Tatas’ motivations too went far beyond therapy.

## Building India’s first tertiary cancer hospital circa 1932–1962

This background provides the context for this historical research, where I aim to show how the founding of Tata Memorial Hospital was motivated by a combination of interests ranging from calls for philanthropic intervention by medical professionals, the economic potential of radium, and state interests in nuclear research. First, I show how the idea of TMH was conceived as part of a longer legacy of philanthro-capitalism in colonial Bombay. I then show how by responding to these different interests the Tatas transformed what was originally conceived as a radium facility within an existing hospital to TMH, a comprehensive cancer hospital (TECS n.d.a.). With the capacity for nuclear research, TMH came to prioritize radium research alongside the treatment of cancer. Responding to the emerging post-colonial interests of the Government of India (GoI), the Tatas strategically manipulated TMH’s governing structure in pursuit of their economic interests in India’s emerging nuclear research economy. TMH’s later transition to the Department of Atomic Energy (DAE) further reinforced the importance of radium at TMH (TECS n.d.a.; Kumar 2018; Phalkey 2013). This history demonstrates not only the actors and interests involved in the validation of radiotherapy, but also it emphasizes how research and education were prioritized while overlooking many issues in access to care. Ultimately, the governance of India’s first tertiary cancer hospital was engineered to allow non-health actors to place stakes in India’s emerging cancer infrastructure and exercise considerable agency in pursuit of their interests.

### Philanthropic health care governance in Bombay and the philanthro-capitalist birth of TMH

Philanthropy in Bombay was commonly administered by civic members of the Parsi community (Palsetia 2005). The Parsis are an ethnic group that migrated from Iran to West India in the eighth century (ibid). Throughout history, the Parsis’ “charity had aided [their] settlement … across Western India providing [an] essential” form of community integration, often enabling them to assume “community leadership” (ibid, 6). Upon the arrival of the British, “the Parsis’ economic wealth and cultural affinity to the British particularly advantaged them in exploiting avenues for socio-political advancement within [a] … new political culture of charity” (ibid, 7). The Parsis were positioned as elite allies to the British, aiding the British government in providing services to citizens through their philanthropy while receiving favorable business conditions in return. In addition to this allyship generating direct business, philanthropy was also made more appealing by the ability to house philanthropic funds in Trusts which received tax deductions from the British government (Palsetia 2003).

Philanthropy also worked to reinforce the power structures of “patron-recipient bonds” between companies and citizens in contexts of vast inequality (Palsetia 2005, p. 202; also see: Piliavsky 2014; Raianu 2017). Specifically, Kumar (2018) identifies Parsi hospitals as creating a space which viewed philanthropy as “a collaborative political community” between citizens, the Parsi elite, and the British government (4). Thus, philanthropically funded Parsi hospitals were both a means of social welfare and a means to reinforce an ideology of the necessity of big businesses. This dual function thereby enabled a “circulation of communal obligation in perpetuity” (Vevaina 2018, p. 1). In this sense, the use of Parsi philanthropy in colonial Bombay can be conceptualized as philanthro-capitalist: a form of philanthropy which goes beyond altruism and specifically acts to support the growth of businesses by philanthropically fostering a communal acceptance of the inequalities that capitalism generates.

The Tatas, the largest Parsi business family in Bombay, were among those to frequently cease philanthropic opportunities to support their capitalist interests. With an increase in biomedical innovation at the turn of the twentieth century, it became apparent to the British government that biomedicine could also benefit populations relevant to British rule. However, health care governance by the British government in colonial Bombay was both “inherently limited and self-limiting [in their approach to disease] … focused on keeping epidemics at bay” (Amrith 2009, p. 8). Rather than the British further investing in health services, the British’s approach to health governance explicitly called upon philanthropic actors to provide health care (ibid). It was within this intersection of philanthro-capitalist norms and biomedical potentials that the philanthropic Tata Trust was motivated to establish TMH in 1941.

TMH was first conceptualized in 1932 when the British appointed Governor of Bombay approached Dorabji Tata, the chairman of the Tata group companies which owned the Tata Trust (TECS n.d.a.) In the context of increasing wartime tensions, the British government had an interest in the research and application of radium (Phalkey 2013). Simultaneously, as detailed in the section on the history of radium, global interests were rising about the role of radium in the treatment of cancer. Specific to Bombay, an increased mortality from cancer among Bombay elites—significantly including Dorabji Tata’s wife’s death to cancer—had heightened the urgency of addressing cancer. Further support for addressing the burden of cancer in Bombay came from the King Edward Memorial Hospital’s 1933 annual report which cited 2,920 cases of cancer and specifically called upon “the great philanthropic public” of Bombay to address the burden (Spies 1935, p. 2). Collectively, these interests made addressing the burden of cancer through the use of radium an ideal philanthro-capitalist project: it would simultaneously strengthen the Tatas’ allyship with the British and respond to the needs of Bombay’s citizens. In other words, by the Tatas philanthropically responding to the burden of cancer, it would reinforce the Tatas’ power between the British government and its citizenry.

### Founding Tata Memorial Hospital

Alongside the global politics of radium, the British government specifically held an interest in radium due to its apparent applications in nuclear research and biomedicine. As the global radium fever spread, radium research slowly revealed its larger industrial potential, specifically in nuclear energy (Phalkey 2013). By 1935, the Tata Trust responded to these interests by launching an investigation into the possibility of establishing a philanthropic ‘radium facility’ in an existing Bombay hospital (TECS n.d.a.). Before beginning the investigation, the Trust was already hesitant about investing into therapeutic radium because they had previously heard from American physicians that radium was ineffective in the treatment of cancer (Tata Trust 1935a; Cantor 2008).

To investigate radium’s potential, the Trust consulted Dr. John Spies. Spies was trained as a physician in the United States. Following his medical training in the 1920s, he attempted to pursue a postgraduate medical training program which would allow him to develop radium for the treatment of cancer. However, Spies’ American supervisors found him to be ‘reckless’ and ‘overly ambitious,’ reflecting the American distrust in radium therapy at the time (Cushings 1930). Failing to find a postgraduate program which would allow him to pursue his radium interests, Spies relocated to China to direct the Tumor Clinic at Peiping Union Medical College, Asia’s first medical center using radium for the treatment of cancer. Spies’ professionally exiled status made him a fitting iconoclast for those desiring legitimization of the role radium could play in the treatment of cancer.

When consulting with the Trust, Spies defended the use of radium by arguing that the cancers seen in India were unique from the United States in that they were often close to the skin, and thus more likely to be treated effectively with radium; specifically, he emphasized the use of brachytherapy (Spies 1935). However, when Spies was pressured by on trustee, he himself even conceded that radium would not be able “to work wonders” (Tata Trust 1935a, p. 1). Further, Spies stressed the urgency of preparing for the rising rates of cancer in India. However, Spies’ data was contradictory. On the one hand, Spies relied upon IMS data to show that cancer types in India were unique; on the other hand, Spies claimed that cancer was highly prevalent in India standing in stark opposition to the “prevailing [IMS] idea that cancer was rare among the natives of India” (Smith and Mallath 2019, 6). Similar to how cancer activists in Madras were ‘ambivalent’ in their arguments presented to the government, Spies’ aggressive championing of radium alongside both acknowledging the limitations of radium and relying upon contradictory epidemiological claims made his advice ambivalent; subsequently, the usefulness of radium remained ambiguous to the Trust (Sivaramakrishnan 2019).

Spies’ arguments also overlooked the emerging body of literature from Indian physicians and instead relied upon reports from the IMS. In contrast to the IMS, Indian physicians not only found an equal prevalence of cancer in India to that seen in the United Kingdom when adjusted for age, but also found that the cancer types in India were similar to those seen in the UK and the USA. Most notably, in 1927 Megaw and Gupta conducted a survey on the epidemiology of cancer in India. “Breast cancer was the most prevalent cancer … followed by mouth cancer” uterine cancer, skin cancer, and stomach cancer (Smith and Mallath 2019, p. 6). Further, Indian physicians suggested in their writings that poor cancer outcomes were often due to little public knowledge of cancer and physicians untrained in identifying cancer (Nath and Grewal 1935; Nath and Grewal 1933). This ultimately questioned the moral legitimacy of using radium for the treatment of cancer when most patients would not receive curative treatment. Opposite of what Spies presented to the Trust, Indian physicians argued that the most common cancer types were not close to the skin and the scientific basis for therapeutic radium at the time was still unfounded.

Nonetheless, when Spies consulted the Trust, he was able to make the idea of using radium for the treatment of cancer appealing. Rather than dwelling on radium science or cancer epidemiology, Spies specifically highlighted how “the future of radium in medicine is promising” and emphasized its “educative value” for industrial development (Spies 1935, p. 2; Tata Trust 1935a, p. 1). In doing this, despite radium’s limitations in treating cancer, Spies was able to frame the use of radium in medicine as something which would benefit the Tatas by providing them with a unique entry point into emerging industries (Tata Trust 1935b). By the end of Spies’ consultation, he was able to transform what was originally conceived as a radium facility in a pre-existing hospital to a full tertiary cancer hospital.

The Tata Trust’s investigations into radium show that TMH was conceived through different forms of philanthro-capitalisms. First, interest in TMH was sparked through aspirations of a traditional colonial philanthro-capitalism which attempted to create “a circulation of communal obligation in perpetuity” (Vevaina 2018, p. 1). Significantly, this philanthropic image of TMH lives on today. When I spoke with TMH staff, patients, and Mumbai residents, the majority recited the popular narrative that TMH was founded because Dorabji Tata’s wife died from cancer and the Trust did a ‘great deed’ for the city. Second, in establishing TMH, the Tata Trust pushed the boundaries of colonial philanthro-capitalism to create a form of philanthro-capitalism which had immediate capitalist aspirations in the industrial applications of nuclear research. However, the Trust’s logic of founding TMH in 1941 with aspirations of radium’s potential business applications does not answer how and why radium-based cancer therapies came to be legitimized. To understand this, it is important to turn to the emerging interests of the soon independent Indian state.

### TMH: a cancer hospital with nuclear research relevance

Soon after TMH was founded in 1941, the Tata Trust needed to realign their philanthro-capitalist aspirations with the Indian Independence Movement. However, Indian politicians soon to be running the post-colonial Indian state from 1947 did not envision local health care philanthropy—such as a single cancer hospital in Bombay—as part of the project of nation building. As Roger Jeffery (1998) has documented, Indian politicians did not “consider long-term goals” in health care planning following independence (105). Instead, the central government largely left the responsibility of health to state governments (Dhillon et al. 2018). However, state governments did not hold equal influence as that of their colonial predecessors. Without the support of the central government, state governments were unable to provide the historical benefits of administering health care through philanthro-capitalism. Because of states’ inability and the central government’s lack of interest in local health care provision, “the culture of … voluntary activity in the field of health witnessed a rapid decline,” specifically in philanthropically funded health care (Amrith 2009, p. 6).

Rather than health care investments, rising independence leaders saw nuclear “research and development in India [as] an immediate necessity” within the context of “the outbreak of WWII [in 1939] and then with the Japanese occupation of Burma” beginning in 1942 (Phalkey 2013, p. 61). The focus of the Indian state on nuclear research and development has been characterized as representing an ideology of ‘scientific-nationalism’ (Roy 2007). Scientific nationalism specifically fostered the idea that India’s “need for science [was] the most urgent and palpable national need” demanding heavy capital investment (ibid, 115). Further, the nuclear research of scientific nationalism was “inserted into fresh forms of patronage by nationalists and philanthropists” providing the opportunity to fill outdated forms of health care philanthro-capitalism (Phalkey 2013, p. 290).

As the scientific nationalist aspirations of the state emerged, Vevaina (2018) has shown how, while philanthropy “remain[ed], what [was] subject to interpretation [was] who [and what was] worthy” of being a beneficiary (Vevaina 2018, p. 248). For the Tatas, Arun Kumar (2018) has shown that the Tata Trust’s philanthropy was pragmatic: it “was attuned to what was practical and not necessarily theoretical, principled, or normative considerations” (17; Markovits 1996, 2008). In other words, to attempt to maintain the benefits formerly awarded to their philanthro-capitalist projects, the Trust’s philanthropy began to cater to the interests of scientific nationalism. For example, by the mid-1940s, the Tata Trust began to reserve 10 percent of their budget for scientific investment (Kumar 2018). With this funding, the Tatas partially funded a National Physical Laboratory, a National Chemical Laboratory, and a National Metallurgical Laboratory (Phalkey 2013). Further, rather than immediately abandoning TMH as an outdated form of philanthro-capitalist health care, instead the Tata Trust identified the potential of TMH’s investment in radium to contribute to the nuclear research interests of scientific nationalism.

When TMH opened in 1941, it held “2000 mgs of radium in solution …. [and a] radon plant … a totally unique facility never seen before in India (TMC 2017, p. 41). The presence of a radon plant within TMH was significant; in the context of a limited global radium supply, rather than navigating complex procurement networks the Tatas had decided to build their own nuclear reactor within the hospital (Creager 2009). As Phalkey (2013) has noted, the nuclear research laboratory was a crucial site for the development of scientific nationalism: “the research laboratory, which had made a small contribution to the development of industry, was now hitched to the defence of the” legitimacy of India’s political and scientific authority within the post-colonial world (66). In other words, despite the small direct impacts radium would provide to cancer, TMH’s radon plant was able to be seen as essential because it supported the project of nation building. To cease the potential of radium, the everyday activities of TMH became deeply embedded within the Tatas’ other projects of nuclear research.

In 1945, the Trust founded the Tata Institute of Fundamental Research (TIFR) for the purposes of nuclear research in collaboration with the Government of Bombay (TECS n.d.b). As TIFR began its research, the radium capacity at TMH was of interest to TIFR to both to strengthen TIFR’s research profile and to use TMH as a political tool to represent the peaceful uses of nuclear research. When TIFR opened, the head of the department of radiophysics at TMH, Dr. Naidu, wrote to the director of TIFR, Dr. Homi Bhabha, in a letter entitled “nuclear investigations at the Tata Memorial Hospital” (Naidu 1945). In this letter, Naidu expressed his excitement that “the Government [of India was] now aware of the vital role that [nuclear] science is playing in this modern world,” and expressed interest to mobilize nuclear research for the purposes of “human betterment” (Naidu 1945, p. 2) Naidu detailed for Bhabha the nuclear facilities that were available at TMH and invited collaboration (ibid).

By Naidu establishing a relationship with Bhabha, this had a significant influence of the direction that TMH would take. Bhabha was the nephew of Dorabji Tata, and also a close friend to the Prime Minister of India, Nehru (Wadia 2009). Bhabha had also completed his PhD in physics at the University of Cambridge and was one of the few Indian physicists at the time to have an international education (ibid). When Bhabha returned to India, as he began his physics research, he attempted to centralize physics research in India in disagreement with many existing Indian physicists (Phalkey 2013). However, with Bhabha’s access to capital through the Tatas and close political connections he was successful and was able to restructure the nuclear research capacity of India almost entirely within TIFR (ibid). Internationally, Bhabha held a number of close connections with researchers, and he also chaired the United Nations Committee on the Peaceful Uses of Atomic Energy where he often used TMH as an example of success (Wadia 2009).

Naidu’s relationship with Bhabha had significant implications for the type of nuclear research which would be conducted at TMH. The immediate years following TIFR’s opening in 1945, Naidu and Bhabha engaged in several research projects and commonly exchanged radioactive substances for comparisons within each other’s laboratories (Bhabha 1945). These collaborations guided the nuclear research conducted in TMH’s department of radiophysics and encouraged Naidu to also focus on the ‘fundamental’ questions of nuclear research.

The influence of TIFR on TMH deepened when Bhabha was asked to appoint Naidu’s replacement in 1947 (TMH 1947). Bhabha’s elite standing in the world of nuclear research allowed him to recruit a physicist from France. Bhabha wrote to Joliot-Curie, daughter of the late Marie Curie credited with discovering radium, to seek advice on a suitable replacement (Bhabha 1947; Kulakowski 2011). Curie recommended her physics student, Mr. Gandy (Curie 1947). However, in the weeks following, Bhabha received a letter from an Indian physicist in Curie’s laboratory who expressed that Gandy had little medical knowledge and the position would be better filled by a medical professional, preferably of Indian origin (Gokhale 1947). However, Bhabha never replied to this letter and continued to recruit Gandy. When Gandy expressed that he was concerned that he would not have ample time to pursue his nuclear research interests at TMH, Bhabha replied to Gandy explaining the close relationship between TMH and TIFR. Bhabha guaranteed Gandy that if he filled the position as head of the radiophysics department at TMH he would be able to collaborate on many projects with TIFR (Bhabha 1947). As Bhabha’s influence grew in the post-colonial state, he was able to appoint additional researchers at TMH for the purposes of nuclear research. In 1948, Bhabha was appointed the chair of the Government of India’s Atomic Energy Commission (AEC) which oversaw all of India’s nuclear capacity; the same year, the AEC funded a unit of TIFR researchers to establish a cell biology laboratory within TMH which would focus specifically on the applications of nuclear research within cell biology (ICRC 1952).

Ultimately, Bhabha’s collaborations with TMH allowed him to further his own nuclear research agenda. Identifying the potential of India’s only radon plant within TMH, Bhabha was interested in recruiting researchers to TMH that would be able to strengthen the nuclear research capacity of India and collaborate with TIFR. For the Tata Trust, TMH’s collaborations with TIFR helped to realize TMH as an essential actor contributing to the nuclear research interests of the post-colonial state’s ideology of scientific nationalism. By positioning TMH within this network, it helped make what were originally only aspirations of the industrial applications of nuclear research brought to the attention of the Trust by Spies slowly become tangible. However, sustaining TMH slowly came to be seen as a strain on the Tata Trust’s financial resources. To assist in realizing the industrial applications of nuclear research, TMH would be ‘gifted’ to the Government of India in 1957.

### The strategic governance of TMH

As the post-colonial state’s interest in nuclear research continued to thrive, the Trust’s philanthropy and other Tata businesses continued to invest in and became reliant upon nuclear research, including that performed between TMH and TIFR. For example, by 1950 Tata Aircraft was using TIFR for “releases of disposals material” which were radioactive (Bhabha 1950, p. 1). Further, J.R.D. Tata, then the chairman of the Tata Group, was collaborating with Bhabha to establish “three separate generations of nuclear power stations” for Tata Incorporated which held investments in the emerging nuclear energy economy (Tata 1958, p. 1). However, J.R.D. Tata was hesitant to fund three separate nuclear power plants due to the large capital resources it would require. Nonetheless, J.R.D. Tata proceeded because unless three separate plants were established, plutonium or uranium would be required to be procured from abroad. “This meant that once we [the Tatas and the GoI] started[ed] the atomic program from borrowed plutonium or uranium [the entire power programme of] India [would] be under international control” bound to the rules of the International Atomic Energy Commission (Bhabha 1958, p. 2). To Bhabha, this was “not a position” the Tatas could “accept” (ibid, 2). In addition to the Tatas’ ongoing investments, a number of universities and GoI offices frequently sent the Tatas invitations asking if they would “‛be interested in starting an industry’” which would be benefitted by their background in nuclear research (Phalkey 2013, p. 237).

While TMH was essential to building the Tatas’ initial expertise in nuclear research through its collaborations with TIFR, the increasing capital demands of the Tatas throughout the 1950s made their annual capital investments to TMH appear burdensome. When I interviewed the daughter of E.J. Borges, former TMH director from the years 1967–1969, she recounted how the Trust was ignorant to the “very labor intensive and … very long treatment [of cancer] so they couldn’t … support this capital-intensive hospital.” Within this context, the Trust invited the GoI to visit TMH in 1951 with the proposal to ‘gift’ TMH to the GoI. However, the GoI, still largely uncommitted to funding health care, was uncertain about accepting the proposal. Nonetheless intrigued by the relevance of TMH to India’s nuclear research project, the GoI upgrading committee visited TMH to learn more about their research and to explore GoI sponsored collaborations in 1952 (ICRC 1952; Matthai 1957; Amrith 2009). Following the visit, the Ministry of Health and Family Welfare (MOHFW) did decide to fund the establishment of the Indian Cancer Research Center (ICRC); the ICRC encompassed both the cell biology unit of the AEC and TMH’s pathology department further strengthening the research capacity of TMH (ibid).

In addition to the GoI’s hesitation to fund a full tertiary cancer hospital, the Tata Trust wanted to arrange a governing agreement with the MOHFW which would allow them to maintain their philanthro-capitalist stakes in nuclear research at TMH. The Tata Trust requested that “the name Tata Memorial Hospital is retained” and the Trust is “entitled to adequate representation on the Governing Board of the Hospital” (Kaur 1956, p. 2). Specifically, the Trust wanted to hold half the seats on the governing board and have the ability to appoint the chairman of the governing board. When the MOHFW pushed back on the conditions of acquiring TMH, the Trust reminded the GoI that “it must be remembered that the founding of this Hospital was originally inspired by the desire to perpetuate an intimate personal memory and those of us who represent the Trust have a responsibility for seeing that the founder’s feelings are not disregarded in any arrangement we agree to now” (Matthai 1956, p. 36). In other words, despite the Trust’s lack of desire to continue funding TMH, they attempted to engineer the conditions of the transfer so both forms of philanthro-capitalism would live on. First, by maintaining the Tata name of the hospital, it ensured that the “intimate personal memory” of the Tatas would continue in a “circulation of communal obligation in perpetuity;” second, by maintaining their seats on the governing board the Trust would be able to continue to monitor the activities of TMH to ensure their alignment with their stakes in nuclear research (ibid; Vevaina 2018, p. 1). The negotiations concluded with all of the Trust’s terms being agreed to except that the chairman of the governing board was to be appointed by the MOHFW beginning from 1960. By 1957, the GoI accepted the proposal for the MOHFW to “take over” TMH (Matthai 1957, p. 1).

However, when the MOHFW took over, TMH received the same amount of funding as all other government hospitals in India, and the quality of research and patient care began to decline (Tata Trust 1957; Lala 1998). Noting the decline in the quality of THM, Bhabha intervened. In 1962, through only three letters addressed to Bhabha, Nehru, and the Tata Trust, Bhabha transferred TMH to the Department of Atomic Energy (DAE) (Choksi 1951; Bhabha 1961; Lala 1998). Compared to the 5 year negotiation process between the Tata Trust and the MOHFW, the ease of the transfer represented Bhabha’s political clout. The transfer of TMH from the MOHFW to the DAE was significant. By that time Bhabha was the director of the DAE and had centralized nuclear research within the DAE to the extent that his mandate over nuclear research was seen as “invincible” (Phalkey 2013, p. 206). As such, positioning TMH within the DAE formalized its necessity to nuclear research; a hospital which had once held associations with the growing body of research and its associated industrial potential was now a critical element validating the necessity of this project. Building on the work of TMH’s radiophysics department, in 1963 the DAE expanded the potential of radium medicine by founding the Radiation Medicine Center (RMC), Asia’s largest center for radiation medicine (BARC 1988). The RMC was focused on the development of new radioisotopes and frequently supplied TMH with radioisotopes for cancer therapy.

Ultimately, although TMH was seen as necessary to the Tatas’ nuclear research expertise, the Tata Trust wanted to refocus their funds away from fundamental nuclear research and towards its immediate industrial applications. The gifting of TMH to the MOHFW reveals a paradox of what were considered ‘the most palpable needs’ of nation building. On the one hand, nation building demanded funding cancer research which was in line with the nuclear research interests of scientific nationalism; while on the other hand, the nuclear research interests of nation building were not necessarily in line with the immediate needs of patients. Further, through the transfer of TMH to the MOHFW, the Trust was able to preserve both forms of its philanthro-capitalism; it maintained the Tata brand reinforcing ‘an intimate personal memory’ and also maintained governing rights to remain involved in TMH’s nuclear research. The final transition of TMH to the DAE solidified its focus on nuclear research and provided the resources for the DAE to further build capacity in radiation medicine. The last section of this article will analyze how philanthro-capitalist and scientific nationalist interests in nuclear research at TMH influenced the practices of cancer therapy and cancer research.

## Cancer therapies and cancer research at TMH circa 1941–1962

It was possible for radiotherapists at TMH to rationalize the use of radium for the treatment of cancer both because of TMH’s radium capacity due to the context outlined above, as well as the late presentation of cancer patients at TMH (Mody 1945; Bhabha 1961). As the nuclear research interests of scientific nationalism became further embedded within TMH, radiotherapeutic practice continued to expand and be seen as legitimate within TMH (Mody 1948b). Beyond TMH, the radiotherapists at TMH attempted to legitimize the practice of radiotherapy for all of India by founding professional organizations such as the Indian Radiological Association (IRA) (Mody 1948b). With the legitimization of radiotherapy through the IRA, other cancer centers followed suit in the expansion of radium medicine programs (Krishnamurthi 2004). However, the capital that the Tata Trust had invested into TMH also provided other opportunities for cancer researchers beyond radiotherapy; drawing on these funds, cancer researchers attempted to make cancer be seen as an important health priority for India (Khanolkar 1950).

### Radiotherapy at TMH

Recalling Spies’ cautionary note that radiotherapy would ‘not be able to work wonders,’ the efficacy and mechanism of action of radiotherapy were still unknown when TMH opened. Nonetheless, rather than attempting to prove its efficacy, the practice and writings of radiotherapists at TMH assumed its efficacy. As early as 1941, TMH’s radiotherapists claimed that radiotherapy was “as an essential and highly effective weapon in the fight against cancer” (Athle 1941, p. 317). However, radiotherapy for the patient was rarely curative, but often a last resort; in contrast to the United States, “cases which [were] ordinarily … surgical problems [had] … come to the radiologist [when] they [were] … often too far advanced” to treat (Mody 1946, p. 3). Because of this, radiotherapists believed that any hope of curative treatment demanded a “radical and thorough approach” (Mody 1948b, p. 1). By 1956, 23,087 patients had been treated with radiotherapy at TMH (Tata Trust 1935a).

While radiotherapy was claimed to be ‘highly effective,’ radiotherapy was practiced under the circumstances of a last-resort form of medical practice (Banerjee et al. 2014; Bhabha 1961; Mody 1945). In other words, the use of radiotherapy can be described as desperate patients and physicians seeking treatment options. Because most patients’ cancers were advanced, the practice of radiotherapy at TMH often saw “widely disappointing” outcomes (Mody 1948b, p. 1). As the TMH transitioned between multiple governing bodies—yet always under the purview of the Tata Trust—medical practice was not held accountable by a health authority. The contrast between radiotherapists’ claim that radiotherapy was ‘highly effective’ while also being ‘widely disappointing’ shows how the use of radiotherapy took place in a largely unregulated context.

The work of the early radiotherapists at TMH provides further insights into radiotherapy’s practice and efficacy for Indian cancer patients (Table 1). Between 1941 and 1956, two notable retrospective studies were conducted looking at outcomes associated with radiotherapy. First, between 1941 and 1948, out of “98 cases of carcinoma esophagus, no patient lived more than 2 years” (Mody 1948a, p. 7). In 1954, out of 122 cases of cervical cancers at TMH, 47 were too advanced, and 15 cases were considered potentially curative and able to be treated with surgery and radiotherapy; of these 15 patients, 2 reached 5-year survival (Mody 1945). Radiotherapy was also applied to bone tumors, breast cancers, and gastro-intestinal cancers where the results continued to be “widely disappointing” (Mody 1946, p. 3). Outside of these studies, little is known about the efficacy of radiotherapy at TMH.

**Table 1:** Number of cases treated with surgery, radiotherapy, or radio-imaging

Year	Surgical operations	Radiotherapy treatments	Radio-imaging
1941	850	932	1845
1942	925	750	2033
1943	1030	966	3886
1944	1099	1176	3520
1945	1003	1131	3486
1946	1083	1389	4808
1947	1175	1382	4665
1948	1184	1406	5309
1949	1128	1738	6390
1950	1165	1406	7108
1951	1358	1677	8029
1952	1828	2290	10,248
1953	2226	2376	11,240
1954	2296	2430	10,888
1955	2359	2438	12,307

Source: Tata Trust 1935a

What is significant about these studies is that they show that while the practice of radiotherapy yielded miniscule therapeutic results, the Tata Trust allocated significant philanthropic funds to strengthen TMH’s radium capacity. The lack of correlation between funding and therapeutic efficacy represents how interests in nuclear research were embedded within cancer therapy and enabled TMH to not address questions over the efficacy of radiotherapy. Instead, the nuclear research interests holding together TMH provided radiotherapists with a provenance which enabled forms of radiotherapeutic experimentation on patients for the benefit of nuclear research. Embedded between the nuclear research interests of the Tata Trust and the late presentation of cancers, radiotherapy became legitimized.

### Radiotherapy Beyond TMH

When the Tata Trust founded TMH, it wanted it to be a model for “centers all over India. In that event, theirs would occupy the proud position of the parent center” (Tata Trust 1935a, p. 1). In this sense, in addition to nuclear research and patient treatment, the Trust created TMH as a hospital which would attempt to lead in the education and research of cancer (Tata Trust 1935b). In an effort to lead in medical education, TMH’s radiotherapists worked closely with the Indian Radiological Association (IRA), a professional body for radiologists across all of India. The IRA held annual conferences and published findings on radium medicine in the Indian Journal of Radiology (Mody 1948b). TMH’s radiotherapists would use these venues to encourage other radiotherapists across India about the validity of using radium in medicine.

In 1947, Dr. Mody, Head of Department of Radiology at TMH, delivered the Presidential Address at the Second Indian Congress of Radiology (ibid). Through this address, Mody articulated to the nation’s radiotherapists the challenges of treating cancer in India and the role of the radiotherapist in this context. Responding to the late presentation and poor outcomes at TMH, Mody stated that he believed that cancer’s “only hope lies in early diagnosis” and encouraged the MOHFW Director-General of Health to administer mobile X-ray units and establish tumor clinics in local hospitals; this recommendation was also made in a government report in 1946 but was never implemented by the MOHFW as this was seen as a state responsibility (Mody 1948b, p. 4; HSDC 1946; Amrith 2009). Mody stated that the challenge of cancer demanded state support for earlier diagnoses, and without it, “radiation [had] … attained its maximum efficiency and nothing further [could] … be expected in that direction” (Mody 1948b, p. 4). However, the hope that Mody did provide to radiotherapists was in the “promising” research area of radioisotopes, a novel area in nuclear medicine utilizing different species of nuclear elements: “such isotopes have proved very valuable as … treatment of leukaemias, thyroid cancers and lymphomas” (Mody 1948b, p. 4).

What is significant about Mody’s advice is that as a radiotherapist he had recognized the necessity of approaches beyond radium medicine to be able to produce better treatment outcomes. Nonetheless, in a context where health investments were made which supported the nuclear research interests of the Tatas rather than the efficacy of cancer treatments, he was unable to mobilize support. Mody responded to these circumstances by encouraging radiotherapists’ improvisation, experimentation, and innovation; in light of all of radiotherapy’s challenges, by closing his address on the ‘promise’ of radioisotopes Mody realigned the duties of radiotherapists with the interests of nuclear research.

The journal and the congresses held by the IRA demonstrated to other hospitals the importance of using radiotherapies in the treatment of cancer, specifically those which were aligned with the state’s nuclear research agenda. In 1956, the Cancer Institute in Chennai (WIA) received a cobalt machine from Atomic Energy of Canada Limited; at the time, Canada was among the world’s top two producers of radium (Krishnamurthi 2004; Cantor 2008). Cobalt machines administered super-voltage radiation: a new form of radiation known to have fewer side effects (ibid). In an interview with the Chairwoman of WIA, V.K. Shantha, whom had personally received the cobalt machine in 1956, she recalled its arrival as a day which changed “our entire meaning. The people from Delhi said, ‘who are these people? What are they trying to do in a hospital?’” WIA was funded through a mix of public and private philanthropic funds, although notably not owned by a large business, and by acquiring India’s first cobalt machine it made WIA nationally relevant. The cobalt machine attracted the attention of Bhabha, who later used the DAE to establish a cesium unit at WIA which further strengthened the centers capacity in radium medicine. In this sense, cancer centers were rewarded for investing in cancer therapies relevant to nuclear research.

### Cancer research and advocacy at TMH

Despite the controversy over radium, the Tata Trust’s sizeable investment into a tertiary cancer hospital was unprecedented and provided physicians and researchers at TMH with opportunities for research. TMH’s funding and network allowed its physicians and researchers to make important advances for understandings of cancer in India. Ultimately, the funding which came with the Tatas’ nuclear research interests also had the effect of building a research capacity which would establish the importance of cancer as a public health problem in India.

One of the most important of these actors was Dr. V.R. Khanolkar. Khanolkar was offered the position of head of the Department of Pathology when TMH opened, but was originally hesitant to join TMH; however, when he heard that the position may go to a non-Indian citizen, Khanolkar felt a ‘national obligation’ to fill the position (Pai 2002). After the Department of Pathology merged with the DAE’s cell biology unit to form the Indian Cancer Research Centre (ICRC), Khanolkar acted as the director of the ICRC from 1952 to 1963. Khanolkar used his positions and the resources of TMH to produce a number of studies which documented the cancer burden and cancer types of India.

When Khanolkar joined TMH, his early work was often in line with the research of the Indian Medical Service which placed a particular focus on cancers unique to India (ICRC 1952). This included cancers such as Dhoti cancer, a cancer associated with a particular article of Kashmiri clothing causing irritation of the skin; Kangri cancer, a cancer associated with the dangling of the pot between the legs to keep warm during winters; Chutta cancer, a cancer associated with smoking by holding the lighted portion of a cigar inside the mouth to keep it lit longer; and penile cancer, specifically the relation that the Hindu practice of not being circumcised had on the incidence of penile cancer (Khanolkar 1945). The majority of this cancer research focused on the socioeconomic and ethnic determinants of the etiology of cancer (ibid; ICRC 1952; Khanolkar 1955). Echoing the interests of the IMS, the focus of this cancer research reflected the colonial ideology that cancer was not prevalent in India unless in novel forms brought on by the unhygienic practices of the colonized peoples (Smith and Mallath 2019).

However, observing the diversity of cancers present at TMH, Khanolkar soon began to question the colonial beliefs that cancers in India were almost exclusively caused by unhygienic practices. As Khanolkar (1951, 1955) developed the department’s research, he began to look at cancers including cancer of the breast, liver cancer, and cancers of the scalp. As the research on cancer types shifted, Khanolkar’s (1944; 1955) research on the etiology of cancer also shifted from socioeconomic and ethnic determinants to determinants including genetics and religion (Sirsat 1945).

Further, Khanolkar was also conscious that a lack of cancer education and oncology training in medical colleges led to a situation where “80 to 90% of … cancer patients reach[ed] the doctors in an advanced stage,” and many did not seek medical attention thus going undocumented (Rai 1953, p. 395). Khanolkar began to study the epidemiology of cancer in Bombay and was the first to adjust for the confounding factors of age and population size in his studies. By doing this, Khanolkar (1950) proved that the burden of cancer in Bombay was equivalent to that seen in New York City when adjusting for age and population. Khanolkar (1951) also proved that “the number of persons suffering from [liver cancer and carcinoma of the scalp were] … proportionately greater than in England or Australia” (57).

From 1950 to 1954 and 1958 to 1962, Khanolkar acted as the President of the International Cancer Research Commission (ICRC; today titled the International Union Against Cancer) (Pai 2002). Khanolkar also served on 4 WHO expert panels, including radiation medicine (ibid). While the President of the ICRC, Khanolkar frequently attempted to rupture the notion that cancer was not prevalent in India. At six separate conferences at the ICRC, Khanolkar commented upon “the general impression that cancer is much rarer in Asiatic and African people than from Anglo-Saxon races;” however, Khanolkar highlighted the global body of research suggesting that cancer rates were similar and frequently cited that studies from “Bombay suggest that the incidence is much the same in Eastern countries as in Western Europe and North America” (Khanolkar 1950, 881; Khanolkar 1951; Khanolkar 1959; Gopal-Ayengar 1951; Rai 1953; Ranadive 1963).

Khanolkar’s research in India and his international representation was significant for how oncologists in Euro-America understood the etiology of cancer. Khanolkar’s studies worked to disprove the IMS’ notion that cancer was not common among the ‘Indian race.’ Khanolkar also had a pan-Indian effect on cancer research and expansion of services. As Sivaramakrishnan (2019) has recounted in Chennai, Khanolkar had visited WIA “to highlight the need for expanding cancer services in Madras” (10). In this sense, the focus of nuclear research at TMH was productive because it provided the funding for actors like Khanolkar to change global perceptions of cancer in India and act as activists for the importance of cancer in health systems planning.

## Conclusion

Contrary to the popular narratives about TMH’s founding, what the history of India’s first tertiary cancer hospital shows is that the founding of TMH and its use of radium was neither a purely altruistic nor a self-evident process. Instead, this article shows how to be able to fund TMH the boundaries between public, philanthropic, and private were extremely porous and mutually contingent. In the context of a nation which still spends disproportionately little on health care in relation to GDP, this suggests that understanding the logics of funding cancer care in India must extend beyond analyses of singular institutional histories and the historical imaginaries they articulate.

In a time when the global use of radium was contested and supplies of radium were hard to come by, the Tata Trust invested large amounts of capital to establish TMH and India’s first radon plant. In doing this, it allowed the Tatas to fulfill two forms of philanthro-capitalism. On the one hand, the Tata Trust was able to be seen as doing a ‘good deed’ for the city and continuing their ‘circulation of communal obligation in perpetuity’ fostering an acceptance of their large business presence. On the other hand, the Tatas founded TMH with larger aspirations for the industrial applications of nuclear research brought to the attention of the Trust by John Spies. First, this reinforces existing conceptualizations of philanthro-capitalism which have suggested that viewing philanthropy as altruistic silences philanthropy’s wider social effects. Yet, further, a second element this article brings forth is philanthro-capitalism’s ability to hold direct ties to capitalist projects, in this case through supporting nuclear research infrastructures.

Framing this history of TMH as embedded within a philanthro-capitalist project then reveals the ways in which histories of philanthro-capitalism are mutually contingent upon the broader interests of nationalism, business, and science. As the Indian Independence movement began to emerge, the traditional business benefits the Tatas received from their philanthropy began to be lost. However, with the nuclear research capacity of TMH, the hospital was able to position itself as an actor in a network which supported the nuclear research interests of post-colonial scientific nationalism. Specifically, TMH’s initial collaborations with TIFR allowed TMH to engage in research activities which would make it a part of this network. As the Tatas’ post-colonial business and philanthropic interests turned towards the immediate profit producing industrial applications of nuclear research, such as nuclear energy, the Tatas came to see TMH as a strain upon their resources. Through TMH’s transfer to the DAE, Bhabha brought TMH back into the network of nuclear research and furthered TMH’s focus on nuclear research through its collaborations with the RMC. It was exactly the ability of the Tata Trust—and its crucial affiliates such as Bhabha—to negotiate the intersections of public, philanthropic, and private interests in cancer which sustained TMH and modified its forms of philanthro-capitalism.

Through the culmination of the philanthro-capitalist and scientific nationalist interests in nuclear research, public, philanthropic, and private actors were able to place stakes in TMH which enabled the contested therapeutic efficacy of radium to appear as a valid form of cancer treatment. Within a context of the late presentation of cancer patients and the hype and hope of radium, scientific definitions of acceptable efficacy and safety became malleable to radiotherapists. Beyond the ethically questionable treatment of late-presenting cancer patients with radium, these intersections also held the productive force to sponsor global leaders in cancer research such as Khanolkar. Importantly, as evidenced by the literature on the use of radium in Euro-America, the ethically contested characteristic of science cannot exclusively be framed as a post-colonial quiddity. In other words, although textualized by (post)colonial philanthropic, economic, and political forces, this history again highlights the potential for scientific appropriation in producing ethical norms.

While Sivaramakrishnan and Mueller have suggested that establishing cancer as a health priority demands a battle against the state and an acceptance of global biomedical paradigms, this history brings a new perspective. In this case, TMH relied upon the colonial and the post-colonial state, and through its involvement with the state and its surplus business capital, the Tatas were able to contravene global biomedical paradigms. This raises further questions for the medical humanities on the relationship between local and global actors in health priority setting; specifically, within which contexts can actors negotiate to exercise power over biomedical paradigms, and at what ethical costs?

Ultimately, comprehending how and why TMH was able to be founded and validate the use of radium for the treatment of cancer works to historicize the context in which cancer has been made legitimate for health systems funding. As India continues to implement public–private partnerships as a form of health systems funding, this history and the others reviewed here provide critical context to understand how the conditions of possibility for cancer patients have been shaped today. Further, understanding these histories can help to imagine new forms of health systems administration which can make the lived realities of patients more commensurable with health systems planning. Doing so enables more nuanced discussions surrounding the lives of patients and the role of health systems in addressing India’s rising cancer burden.

### Archives Consulted

Tata Central Archives, Pune, Maharashtra, India.

Tata Institute of Fundamental Research, Mumbai, Maharashtra, India.

Tata Memorial Centre Library, Mumbai, Maharashtra, India.

The British Library, London, United Kingdom.

The Cancer Institute, Chennai, Tamil Nadu, India, Personal Communication and Personal Records.

Yale University Archives, Virtual Communication.
